# A Case of Unilateral Sixth Cranial Nerve (CN VI) Avulsion in the Prepontine Cistern After Severe Head Trauma

**DOI:** 10.7759/cureus.37486

**Published:** 2023-04-12

**Authors:** Thomas Kent, Vikash Sinha, Mehmet Albayram

**Affiliations:** 1 Radiology, University of Florida College of Medicine, Gainesville, USA

**Keywords:** abducens palsy, cranial nerve 6 palsy, neuro mri, severe head trauma, cn vi

## Abstract

The patient was a 46-year-old woman who presented with right sixth cranial nerve (CN VI) palsy following severe head trauma secondary to a motor vehicle collision one month prior. In this case report, we aim to add to the literature an additional example of unilateral CN VI avulsion as visualized by MRI secondary to head trauma. 3D T2 MRI was used to visualize the CN VI avulsion. CT was also used in the evaluation of head trauma. In our view, the force trajectory of the patient’s impact with the vehicle dashboard, as evidenced by the right occipital lobe fracture, explains the etiology of the unilateral right CN VI avulsion. The combination of clinical and imaging findings was central to the analysis of this case.

## Introduction

The incidence of unilateral sixth cranial nerve (CN VI; abducens) palsy following head trauma is reported to be between 1 and 2.7% [1]. Of the ocular motor cranial nerves, the abducens nerve is the most susceptible to neuropathy, and trauma causes around 15% of abducens nerve palsies [2]. The complex and extended intracranial course of the abducens nerve makes it susceptible to traumatic injury, including avulsion. A few case reports have documented imaging findings suggestive of unilateral or bilateral CN VI avulsion following severe head trauma [3, 4]. We report a patient with right lateral gaze palsy following head trauma from a motor vehicle accident. 3D, T2 thin-slice MR evaluation revealed an avulsion of the right abducens nerve in the cisternal segment with a thickened, unattached fragment present in the prepontine cistern.

## Case presentation

**Figure 1:**
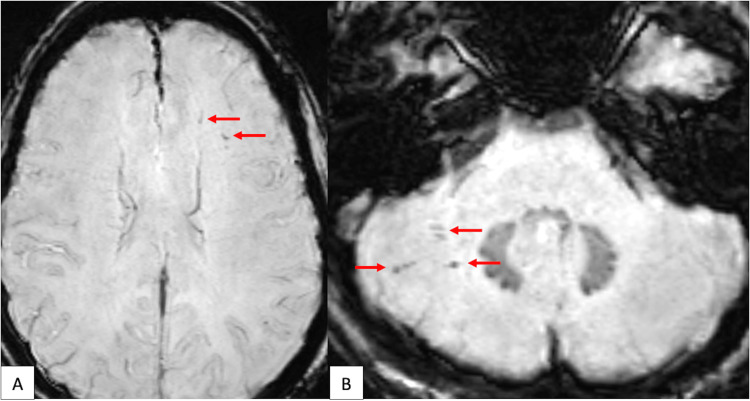
Axial susceptibility-weighted images of the left frontal lobe (A) and right cerebellum (B). Panels A and B demonstrate diffuse axonal injury in the left frontal lobe and right cerebellum, respectively.

**Figure 2:**
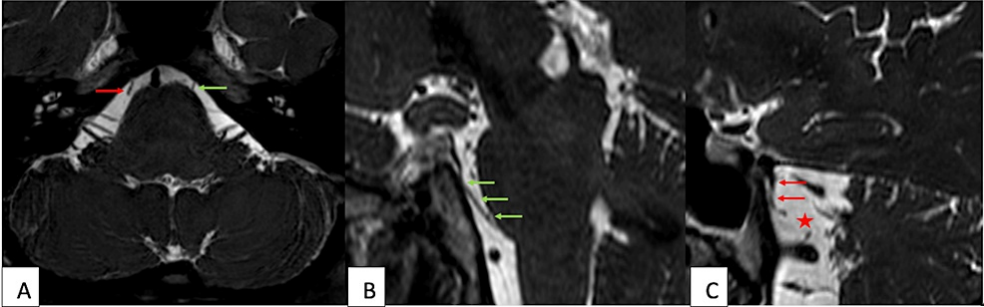
Axial (A) and sagittal oblique (B, C) 3D T2-weighted images of the posterior fossa. Panels A and B demonstrate the usual course of the intact left CN VI from the brainstem through the cisternal segment into Dorello’s canal (green arrows). In Panels A and C, the proximal cisternal segment of the right CN VI (red arrows) is not seen, and the distal cisternal segment is thickened, redundant, and discontinuous with the brainstem. A red star indicates the gap between the visualized cisternal segment and the brainstem. CN VI: Sixth cranial nerve.

**Figure 3:**
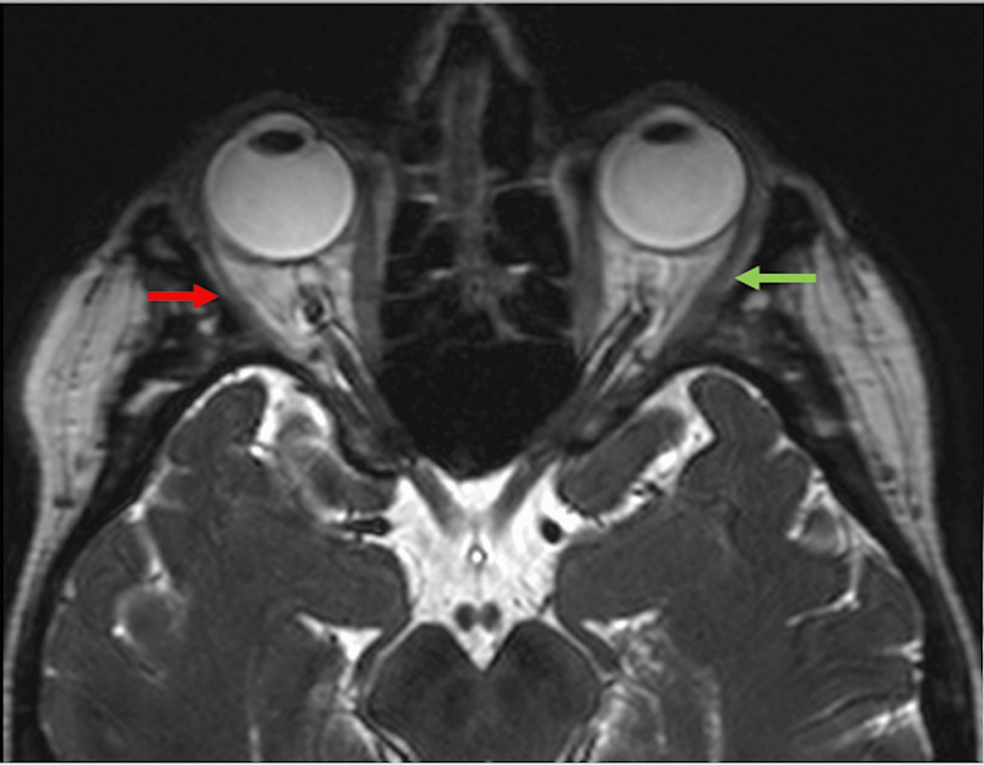
Axial 3D T2-weighted image of a bilateral orbit. The image demonstrates atrophy of the right lateral rectus muscle (red arrow) compared to the normal left lateral rectus muscle (green arrow).

The patient was a 46-year-old woman with no significant medical history who sustained head trauma following a motor vehicle collision. At the presentation, the Glasgow Coma Scale was 14 due to confusion. The patient was noted to have a lateral gaze palsy on the right. Inpatient CT revealed intracranial (subarachnoid/subdural) bleeding with a mildly displaced acute fracture involving the right occipital condyle, left lateral nondisplaced fracture of C1, and fracture of the left medial clavicle. Subsequent inpatient CT a few days later revealed mild, asymmetric widening of the right petrous-sphenoid fissure with stable, small-volume intracranial blood products. Magnetic resonance (MR) and MR venogram studies of the brain were obtained, demonstrating diffuse axonal injury with tiny microhemorrhages in the left frontal and parietal lobes and right cerebellum (Figure 1). The posterior fossa and the course of CN VI were not well evaluated at this time as thin slice T2-weighted images of the area were not obtained in the standard MR brain protocol. The remainder of her inpatient course was uneventful, and she was discharged with instructions for outpatient specialist follow-up. At a follow-up outpatient otolaryngology appointment 35 days later, the patient exhibited persistent lateral gaze palsy. MRI of the cavernous sinus and skull was performed to evaluate for a potential etiology, including thin-slice 3D T2-weighted images through the skull base, cavernous sinus, and posterior fossa. This MR demonstrated the left CN VI taking a normal course through the prepontine cistern into Dorello’s canal and the left cavernous sinus. The proximal cisternal segment of the right CN VI was not seen, and the distal cisternal segment was noted to terminate abruptly in the prepontine cistern with no connection to the brainstem (Figure 2). This study also demonstrated the atrophy of the right lateral rectus muscle (Figure 3).

## Discussion

The abducens nerve innervates the ipsilateral lateral rectus muscle, which is a muscle that abducts the eye or moves it laterally [5]. Several imaging case reports have previously described CN VI avulsion, both unilateral and bilateral, following trauma [3, 4, 6]. Post-trauma CN VI palsy, in general, has been relatively more greatly reported [7]. This case report presents an additional case of unilateral CN VI avulsion secondary to head trauma. Of all the cranial nerves, the abducens nerve has the longest subarachnoid course as its S-shaped pathway takes it vertically at the brainstem from the lower pons along the clivus to then pierce the dura and enter Dorello’s canal. The nerve then enters the posterior aspect of the cavernous sinus and exits through the superior orbital fissure to innervate the lateral rectus muscle. The abducens nerve is susceptible to injury related to a number of etiologies, including neoplastic, traumatic, aneurysmal, and ischemic [7], with microvascular ischemia being the most common [5]. Most abducens nerve palsies are unilateral and acquired [5], with etiology further characterized by the implicated section of the nerve, such as the section within and proximal to the brainstem, the vertical course over the clivus, at the petrous apex under the petroclinoid ligament, in the cavernous sinus, or orbit [7]. The clinical presentation of a unilateral nerve palsy is an esotropic eye. This eye points inward toward the nose due to the unopposed adducting medial rectus muscle, which would normally be opposed by the abducting tonic force of the lateral rectus [5]. For our case, the right CN VI is suspected of having been in the path of the motor vehicle collision (MVC) force vector, which extended from the left frontal lobe superiorly to the right cerebellum and occipital condyle inferiorly, leading to fracture of the right occipital condyle and diffuse axonal injury along the vector. High-energy, blunt force is typically required to fracture the occipital condyle, and such fractures are relatively uncommon [8].

Given the long, complex path of the abducens nerve, the mechanism of injury leading to avulsion is suspected to be related to contusion or stretching of the nerve at some point along its course [3, 9]. While CN VI palsies can be characterized by both etiology and section of nerve implicated, such as various brainstem syndromes, the cavernous sinus syndrome, or diseases that increase intracranial pressure (ICP) leading to stretching of CN VI in the subarachnoid space, a specific section of CN VI vulnerable to severe head trauma has not yet been identified. Interestingly, of the few reported MRI cases of abducens avulsion due to head trauma, both bilateral and unilateral [3, 4, 6], the section of nerve implicated in each case was the cisternal segment between the nerve’s exit from the brainstem and its entrance into Dorello’s canal. This section could be particularly vulnerable to mechanical stress, such as stretching due to upward or downward displacement, as it is tethered at both ends [3, 6]. Post-mortem analysis of traumatic bilateral abducens palsy, though not specifically avulsion, found relatively greater injury, including perineural hemorrhage and edema of CN VI at the dural entry point and the portion above the petrous apex rather than beneath the petroclinoid ligament. These structures were described as rigid structures that could act to enable stretching against the dural entry point of CN VI during upward cervical trauma or compression against the petrous apex during downward brain displacement [10]. As others have noted, evaluating other cranial nerves, such as CN VII and CN VIII, is necessary when presented with a case of CN VI palsy with avulsion because of head trauma due to these nerves’ proximity to CN VI at the brainstem [3]. While CT can be used to identify associated skull bone fractures and/or intracranial hemorrhage [3], CT can also demonstrate the absence of findings [6]. MRI is an essential tool in evaluating severe head trauma with CN VI palsy as it can potentially identify CN VI avulsion and aid in diagnosis. Specifically, thin slice 3D T2-weighted images through the posterior fossa make identifying and evaluating these structures possible and should be sought when exam findings raise concern for injury in this area. The prognosis for spontaneous abducens nerve palsy, whether due to compression or stretching, is highly variable, with reported resolution ranges between 12 and 73%. Unfortunately, limited evidence supports the resolution or surgical repair of a completely avulsed abducens nerve [11].

## Conclusions

We report a case of unilateral CN VI palsy following severe head trauma. MRI 3D T2 demonstrated avulsion of right CN VI in the prepontine cistern, which appears to be a vulnerable location for CN VI avulsion following head trauma in the existing imaging literature. While the resolution of spontaneous CN VI palsies following trauma is possible following stretching or compression of the nerve, there is limited evidence of the resolution of CN VI palsy following a complete nerve avulsion.

## References

[1] Arias MJ (1985). Bilateral traumatic abducens nerve palsy without skull fracture and with cervical spine fracture: case report and review of the literature. Neurosurgery.

[2] Pihlblad MS, Demer JL (2014). Hypertropia in unilateral isolated abducens palsy. J AAPOS.

[3] Yamasaki F, Akiyama Y, Tsumura R (2016). Post-traumatic unilateral avulsion of the abducens nerve with damage to cranial nerves VII and VIII: case report. NMC Case Rep J.

[4] Azad TD, Veeravagu A, Corrales CE, Chow KK, Fischbein NJ, Harris OA (2016). Abducens nerve avulsion and facial nerve palsy after temporal bone fracture: a rare concomitance of injuries. World Neurosurg.

[5] Thomas C, Dawood S (2021). Cranial nerve VI palsy (abducens nerve). Dis Mon.

[6] Ravindran K, Lorensini B, Gaillard F, Kalus S (2017). Bilateral traumatic abducens nerve avulsion: a case series and literature review. J Clin Neurosci.

[7] Azarmina M, Azarmina H (2013). The six syndromes of the sixth cranial nerve. J Ophthalmic Vis Res.

[8] Leone A, Cerase A, Colosimo C, Lauro L, Puca A, Marano P (2000). Occipital condylar fractures: a review. Radiology.

[9] Lopes BS, Amaral LL, Bezerra HG, Rogério RM, Zambon AA (2011). Bilateral traumatic avulsion of abducens nerve. Arq Neuropsiquiatr.

[10] Sam B, Ozveren MF, Akdemir I, Topsakal C, Cobanoglu B, Baydar CL, Ulukan O (2004). The mechanism of injury of the abducens nerve in severe head trauma: a postmortem study. Forensic Sci Int.

[11] Serio F, Choi J, Mccague A (2019). Bilateral abducens nerve palsy after closed head trauma without acute intracranial pathology. J Emerg Trauma Shock.

